# Supplementary material for: Resolving the full spectrum of human genome variation using Linked-Reads
Source: Genome Res. 2019 Apr;29(4):635–45. doi: 10.1101/gr.234443.118 (PMC6442396; doi:10.1101/gr.234443.118)
Supplement: Supplemental Material [file supp_gr.234443.118_Supplemental_longranger-2.2.zip › longranger-2.2.tar.bz2!/lr2.2-public/lib/stan/cmdstan/stan/doc/api/html/cmd__line_8hpp_source.html]

Stan: src/stan/io/cmd\_line.hpp Source File


|  |  |
| --- | --- |
|  | Stan  2.5.0  probability, sampling & optimization |


- Main Page
- Related Pages
- Namespaces
- Classes
- Files

- File List
- File Members

All Classes Namespaces Files Functions Variables Typedefs Enumerations Enumerator Friends Macros Pages

- src
- stan
- io

cmd\_line.hpp

Go to the documentation of this file.

1 #ifndef STAN\_\_IO\_\_CMD\_LINE\_HPP

2 #define STAN\_\_IO\_\_CMD\_LINE\_HPP

3

4 #include <map>

5 #include <ostream>

6 #include <set>

7 #include <string>

8 #include <sstream>

9 #include <vector>

10

11 namespace stan {

12

13  namespace io {

14

27  void pad\_help\_option(std::ostream\* o,

28  const std::string& option = "",

29  unsigned int width = 20) {

30  if (!o) return;

31  \*o << " " << option;

32  int padding = width - option.size();

33  if (padding < 2) {

34  \*o << std::endl;

35  padding = width + 2; // 2 is

36  }

37  for (int i = 0; i < padding; ++i)

38  \*o << ' ';

39  }

40

49  void print\_help\_helper(std::ostream\* o,

50  const std::string& key\_val,

51  const std::string& msg,

52  const std::string& note = "") {

53  if (!o) return;

54  pad\_help\_option(o,key\_val);

55  \*o << msg

56  << std::endl;

57  if (note.size() > 0) {

58  pad\_help\_option(o,"");

59  \*o << " (" << note << ")"

60  << std::endl;

61  }

62  \*o << std::endl;

63  }

64

74  void print\_help\_option(std::ostream\* o,

75  const std::string& key,

76  const std::string& value\_type,

77  const std::string& msg,

78  const std::string& note = "") {

79  std::stringstream ss;

80  ss << "--" << key;

81  if (value\_type.size() > 0)

82  ss << "=<" << value\_type << ">";

83  print\_help\_helper(o,ss.str(),msg,note);

84  }

85

113  class cmd\_line {

114  private:

115  std::string cmd\_;

116  std::map<std::string,std::string> key\_val\_;

117  std::set<std::string> flag\_;

118  std::vector<std::string> bare\_;

119  void parse\_arg(const std::string& s) {

120  if (s.size() < 2

121  || s[0] != '-'

122  || s[1] != '-') {

123  bare\_.push\_back(s);

124  return;

125  }

126  for (size\_t i = 2; i < s.size(); ++i) {

127  if (s[i] == '=') {

128  key\_val\_[s.substr(2,i - 2)] = s.substr(i + 1,s.size() - i - 1);

129  return;

130  }

131  }

132  flag\_.insert(s.substr(2,s.size()));

133  }

134  public:

142  cmd\_line(int argc, const char\* argv[])

143  : cmd\_(argv[0]) {

144  for (int i = 1; i < argc; ++i)

145  parse\_arg(argv[i]);

146  }

147

155  std::string command() {

156  return cmd\_;

157  }

158

165  bool has\_key(const std::string& key) const {

166  return key\_val\_.find(key) != key\_val\_.end();

167  }

168

188  template <typename T>

189  bool val(const std::string& key, T& x) const {

190  if (!has\_key(key))

191  return false;

192  std::stringstream s(key\_val\_.find(key)->second);

193  s >> x;

194  return true;

195  }

196

203  bool has\_flag(const std::string& flag) const {

204  return flag\_.find(flag) != flag\_.end();

205  }

206

212  size\_t bare\_size() const {

213  return bare\_.size();

214  }

215

229  template <typename T>

230  bool bare(size\_t n, T& x) const {

231  if (n >= bare\_.size())

232  return false;

233  std::stringstream s(bare\_[n]);

234  s >> x;

235  return true;

236  }

237

238

245  void print(std::ostream& out) const {

246  out << "COMMAND=" << cmd\_ << '\n';

247  size\_t flag\_count = 0;

248  for (std::set<std::string>::const\_iterator it = flag\_.begin();

249  it != flag\_.end();

250  ++it) {

251  out << "FLAG " << flag\_count << "=" << (\*it) << '\n';

252  ++flag\_count;

253  }

254  size\_t key\_val\_count = 0;

255  for (std::map<std::string,std::string>::const\_iterator it = key\_val\_.begin();

256  it != key\_val\_.end();

257  ++it) {

258  out << "KEY " << key\_val\_count << "=" << (\*it).first;

259  out << " VAL " << key\_val\_count << "=" << (\*it).second << '\n';

260  ++key\_val\_count;

261  }

262  size\_t bare\_count = 0;

263  for (size\_t i = 0; i < bare\_.size(); ++i) {

264  out << "BARE ARG " << bare\_count << "=" << bare\_[i] << '\n';

265  ++bare\_count;

266  }

267  }

268

269  };

270

271  // explicit instantation for std::string to allow for spaces

272  // in bare\_[n]

273  template <>

274  bool cmd\_line::bare<std::string>(size\_t n, std::string& x) const {

275  if (n >= bare\_.size())

276  return false;

277  x = bare\_[n];

278  return true;

279  }

280

281  // explicit instantation for std::string to allow for spaces

282  // in key\_val\_

283  template <>

284  bool cmd\_line::val<std::string>(const std::string& key, std::string& x) const {

285  if (!has\_key(key))

286  return false;

287  x = key\_val\_.find(key)->second;

288  return true;

289  }

290

291

292  }

293

294 }

295

296 #endif

stan::io::pad\_help\_option

void pad\_help\_option(std::ostream \*o, const std::string &option="", unsigned int width=20)

Print help option with padding.

**Definition:** cmd\_line.hpp:27

stan::io::cmd\_line::val

bool val(const std::string &key, T &x) const

Returns the value for the key provided.

**Definition:** cmd\_line.hpp:189

stan::io::cmd\_line::has\_key

bool has\_key(const std::string &key) const

Return true if the specified key is defined.

**Definition:** cmd\_line.hpp:165

stan::io::cmd\_line::cmd\_line

cmd\_line(int argc, const char \*argv[])

Construct a command-line argument object from the specified command-line arguments.

**Definition:** cmd\_line.hpp:142

stan::io::cmd\_line

Parses and stores command-line arguments.

**Definition:** cmd\_line.hpp:113

stan::io::cmd\_line::has\_flag

bool has\_flag(const std::string &flag) const

Return true if the specified flag is defined.

**Definition:** cmd\_line.hpp:203

stan::io::print\_help\_helper

void print\_help\_helper(std::ostream \*o, const std::string &key\_val, const std::string &msg, const std::string &note="")

Prints single print option to output ptr if non-null.

**Definition:** cmd\_line.hpp:49

stan::io::cmd\_line::bare\_size

size\_t bare\_size() const

Return the number of bare arguments.

**Definition:** cmd\_line.hpp:212

stan::io::cmd\_line::print

void print(std::ostream &out) const

Print a human readable parsed form of the command-line arguments to the specified output stream...

**Definition:** cmd\_line.hpp:245

stan::io::cmd\_line::bare

bool bare(size\_t n, T &x) const

Returns the bare argument.

**Definition:** cmd\_line.hpp:230

stan::io::print\_help\_option

void print\_help\_option(std::ostream \*o, const std::string &key, const std::string &value\_type, const std::string &msg, const std::string &note="")

Prints single print option to output ptr if non-null.

**Definition:** cmd\_line.hpp:74

stan::io::cmd\_line::command

std::string command()

Returns the name of the command itself.

**Definition:** cmd\_line.hpp:155


---

[ Stan Home Page ]

*© 2011–2014,
Stan Development Team.*
